# 3D-printed mirror-less helicity preserving metasurface “mirror” for THz applications

**DOI:** 10.1515/nanoph-2024-0711

**Published:** 2025-06-09

**Authors:** Jiaruo Yan, Ioannis Katsantonis, Savvas Papamakarios, Panagiotis Konstantakis, Michael Loulakis, Thomas Koschny, Maria Farsari, Stelios Tzortzakis, Maria Kafesaki

**Affiliations:** Institute of Electronic Structure and Laser, Foundation for Research and Technology Hellas (FORTH), N. Plastira 100, 70013 Heraklion, Greece; Department of Materials Science and Engineering, University of Crete, 70013, Heraklion, Greece; Department of Physics, National and Kapodistrian University of Athens, GR-15784 Athens, Greece; Ames Laboratory and Department of Physics and Astronomy, Iowa State University, Ames, IA, 50011, USA

**Keywords:** metasurfaces, THz radiation, 3D-printed metasurfaces

## Abstract

Stimulated by seminal works on generalized reflection and refraction laws, metasurfaces have evolved to a highly promising research direction, as they allow a multitude of different functionalities by optically thin wave-control elements/structures. Among them, structures functioning in the low THz regime are of extensive research interest, due to their high potential in communication and sensing applications. In this paper we propose a simple THz metasurface design that exhibits ideally perfect cross-polarized reflection for linear polarization and perfect helicity-preserving reflection with a geometric (Pancharatnam–Berry) phase for circular polarization, without the presence of any back-reflector. Numerical calculations demonstrating the structure response are justified by analytical models, which provide physical insights on this response. The designed metasurfaces are fabricated using the direct laser writing 3D-printing technology, metallized with electroless silver plating, and are characterized by THz time domain spectroscopy, with the experimental results validating the corresponding theoretical ones. Applications including beam steering and focusing, exploring the Pancharatnam–Berry phase, are also demonstrated numerically. Besides those applications, the helicity preserving mirror response of our metasurfaces can be valuable, among others, also in molecular chirality sensing applications, an issue that is also highlighted here.

## 1 Introduction

Prompted by the seminal works by Federico Capasso on generalized reflection and refraction laws and their practical implementation via phase-gradient metasurfaces [1], [2], [3], metasurfaces have evolved into the most promising and ambitious branch of the metamaterials research. The associated possibility of enormous wave control by just a thin layer of electromagnetic (EM) scatterers/resonators [4] has led to a vast expansion of the interest and the research efforts on metasurfaces. Many different structures have been proposed, targeting different novel optical properties and different applications, in a variety of application domains [5], [6], [7], [8].

Among them a strong research effort has been devoted to investigation of metasurfaces for polarization related phenomena and applications. Such metasurfaces promise advanced solutions for polarization control elements, like polarization filters [9], polarizers [10], [11], [12], [13], wave-plates [14], twistronics [15] etc. (e.g. small size, high efficiency), but also enable novel effects and possibilities for light manipulation [16], [17], [18], [19], [20]. The latter is more pronounced in the case of circular polarization, where metasurfaces have allowed strong chiral light–matter interactions [21], [22], [23], [24], [25], [26], photonic spin-Hall effect [27], optical spin–orbit coupling [28], [29], etc. Regarding the chiral light–matter interactions [30], [31], metasurfaces acting as helicity preserving mirrors [32], [33] have been shown, among others, to provide advanced solutions in molecular chirality sensing and enantiomer identification [34], [35], [36], [37] (see also Supplementary Information), an issue extremely critical for life sciences and pharmacology.

Another prominent example relevant to circular polarization control is the geometric (Pancharatnam–Berry) phase metasurfaces [38], [39], [40], [41], [42], which enable different functionalities (e.g. beam steering, focusing [39], [43]) by employing and simply rotating the meta-atoms relative to the lattice.

In this work, motivated by the above mentioned developments, we demonstrate a very simple metasurface element (meta-atom) able to create, in a metasurface configuration, different functionalities for both linearly and circularly polarized waves. For linear polarization our proposed metasurface can lead to perfect cross-polarized reflection, without the addition of any back reflector [44], [45], i.e. leaving the rest of the spectrum available for transmission-related functionalities. For circular polarization it leads to perfect co-polarized reflection, acting as helicity preserving mirror for both right-handed circularly polarized waves (RCP) and left-handed circularly polarized waves (LCP). Moreover, this perfect co-polarized reflection component is equipped with a geometric (Pancharatnam–Berry) phase, giving the ability to create phase gradient metasurfaces for anomalous reflection and focusing of the reflected waves, as we also demonstrate in this work. Finally, the helicity-preserving mirror response of our metasurface leads to fields of high and highly-uniform optical chirality in the vicinity of the meta-atoms; this response promises enhanced molecular chirality sensing potential, exploitable either through placing chiral molecules on top of the metasurface, or by using the metasurface for the formation of chiral cavities [37].

**Figure 1:**
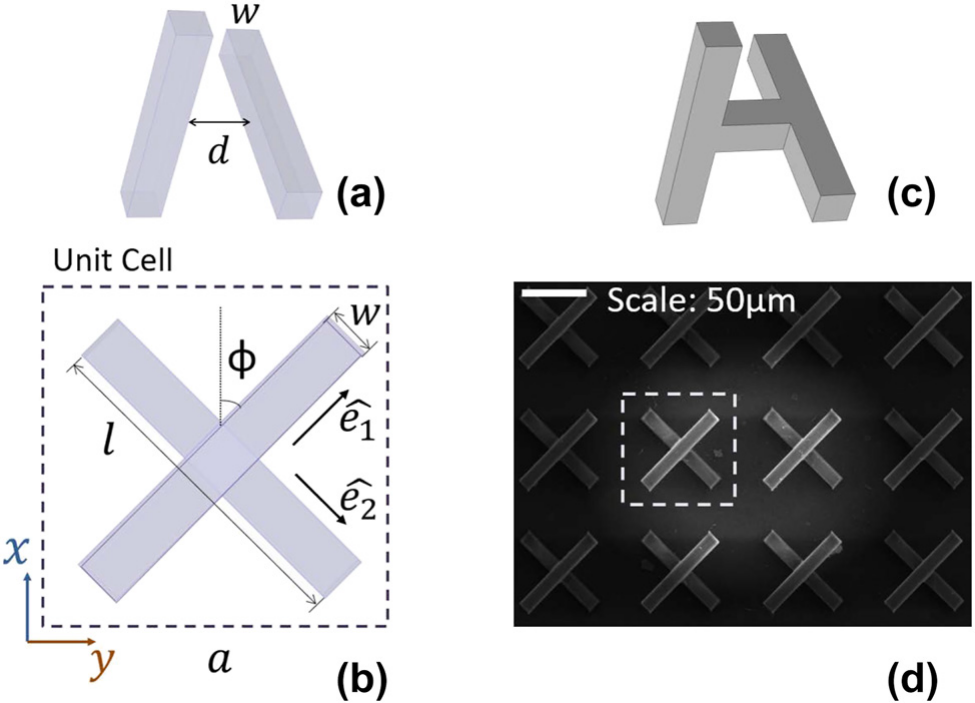
Schematic showing the unit cell of the two-bar structure. (a) Gives a perspective view, showing the width, *w*, of the bar (of square cross-section), and the vertical distance between the two bars, *d*. (b) Gives a front view, showing bar length *l*, and unit cell size *a*. The two bars are orthogonal, and oriented in a way that they are diagonally aligned with the square unit cell, *ϕ* = *π*/4. (c) Shows a vertical bar connecting the two functional bars, resembling the meta-atom in the simulated and fabricated sample. (d) Shows a SEM image (top view) of the fabricated metasurface on a silicon substrate; at the top left corner is a 50 μm scale bar. One unit cell is marked with a dashed square.

Our proposed meta-atom is formed by a pair of perpendicular resonant metallic bars (often called here functional bars), as shown in Figure 1. To enable a dielectrics-free fabrication of the structure, e.g. by direct laser writing (accompanied by silver plating), the two metallic bars are connected, center-to-center, by a third, vertical bar, aiming to maintain a critical distance between the functional bars, not affecting though the structure’s electromagnetic response (at least for normal and small-angle incidence; this is due to the symmetry of the structure). The meta-atom unit cell configuration, as shown in Figure 1, is superimposable to its mirror image through translations and rotations, eliminating any chiral or bianisotropic response, differently to similar systems that have been discussed in the bibliography [46], [47], [48], while a critical factor for its response, as we will show later on, is the distance between the functional bars, as this distance determines the relative reflection phase of the two orthogonal components of the impinging electromagnetic waves. We have to note here that, although our structure has not been investigated in the context of metasurface research, similar structures or structures based on the same principle have been proposed for polarization control in microwave engineering [49], [50], [51]. They are, though, of slightly different geometries (including broken symmetries) or targeting CP selectivity, unlike the response and functionalities discussed here and without the multi-functionality character of our proposed structure.

Although the here presented analysis and demonstration of the response of the structures/metasurfaces formed by the two-bar meta-atom is mainly theoretical, corresponding experimental data are also provided, aiming to validate the achieved theoretical response. The experimentally measured metasurfaces are fabricated by multiphoton lithography, which allows the fabrication of 3D structures with high repeatability, followed by electroless silver plating for the creation of the metal-covered meta-structure. The structures are of micrometer scale targeting operation in the low THz region. Their electromagnetic response, under linearly polarized incident wave, is investigated using THz time domain spectroscopy (THz-TDS).

The THz operation of our structures is of great importance regarding applications, as the THz frequency band is valuable for both future wireless communications and advanced sensing applications, whilst still lacking efficient and versatile wave-control components and devices. To our knowledge, devices with the level of control of reflection as promised by our metasurfaces are still lacking in the THz region. Moreover, the chirality sensing capabilities of our structures (see Supplementary Information) make them suitable for THz chirality sensing applications, an issue highly unexplored to date. Furthermore, the 3D-printing fabrication approach allows in our case, among others, dielectrics-free fabrication, avoiding the dielectric losses inherent in most of the commonly used THz materials (coming, e.g., from phononic resonances).

The rest of the paper is organized as follows. Section 2 describes the structure/metasurface geometry and demonstrates numerically its response for both linearly and circularly polarized waves. This analysis is given for substrate-free metasurfaces, as to avoid any substrate impact on the structure reflection properties. (Note that direct-laser writing process gives the ability to minimize or eliminate the impact of the substrate, by elevating the structure from the substrate, e.g. by adding vertical “legs” [52].) Moreover, Section 2 provides a simple physical model of the structure’s performance, illustrating its dependence on system parameters. Section 3 discusses the experimentally obtained structures and their response, comparing with corresponding numerical data and validating the analytical and numerical study. Section 4 presents and demonstrates the Pancharatnam–Berry phase response of the structure and the achievable beam steering and focusing capabilities. Finally, given that in the optical region displacement currents can undertake the role of the conduction currents, Section 5 shows that our structure’s reflection response can be successfully transferred to the optical region by using high-index dielectric bars as electromagnetic resonator elements.

## 2 Two-bar resonator and perfectly reflective metasurface

### 2.1 Geometry of the meta-atoms

The two-bar structure we propose is shown in Figure 1, with the two identical metallic bars (of square cross-section with *w* = 12 μm and of length *l* = 80 μm) orthogonal to each other, and their centers vertically (along *z*) shifted by a distance *d* = 24 μm. The two bars are placed along the diagonals of a square unit cell of side *a* = 96 μm. In the actual (numerically investigated) and fabricatable (via direct laser writing) structure, a vertical bar (of length *d*) connects the horizontal (functional) bars from center to center, as shown in Figure 1(c). This vertical bar does not contribute though significantly to the system electromagnetic response (at least for normal and small angle incidence).

In the next sub-sections we investigate, both theoretically and numerically, the response of a metasurface composed of two-bar unit cells as the one of Figure 1(b) to both linearly polarized (LP) and circularly polarized (CP) waves. The numerical investigation is performed using CST Studio Suite software, considering normally incident waves (i.e. along *z* direction) and periodic (unit cell) boundary conditions along *x* and *y* directions. The bars’ conductivity is taken as 5.8 × 10^7^ S/m in the calculations.

### 2.2 Linear polarization response

**Figure 2:**
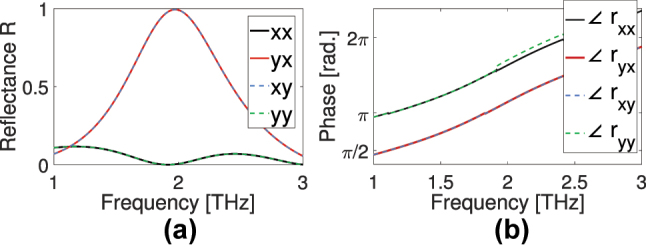
Numerically calculated reflective response of the metasurface to normally incident linearly polarized waves. (a) Is the reflectance (amplitude-squared of the reflection coefficient, *R* = |*r*|^2^). Cross-polarized cases are *R*_*yx*_ (red) and *R*_*xy*_ (blue dashed) Co-polarized reflectance *R*_*xx*_ (black) and *R*_*yy*_ (green-dashed). Perfect cross-polarized reflection is shown near 2 THz. (b) Is the phases of the reflection coefficients in radians.

In Figure 2 we show the structure reflection response for linearly polarized incident waves, for the distance *d* between the two bars being 24 μm. As can be seen in Figure 2(a), close to 2 THz, perfect cross-polarized reflection response is observed for both polarizations (*R*_*yx*_ = |*r*_*yx*_|^2^; in red; *r*_*yx*_: reflection coefficient; and *R*_*xy*_ is blue dashed), where simultaneously the co-polarized reflection *R*_*xx*_ (black), and *R*_*yy*_ (green dashed) are suppressed. The phases of the simulated reflections are plotted in Figure 2(b). As we have *ϕ* = *π*/4 illustrated in Figure 1(b), and the two bars diagonally aligned with the unit cell, the reflection phases are the same for both polarizations, with a phase difference of *π*/2 between co- and cross-polarized reflections. It is noteworthy that the high cross-polarized reflection response is highly dependent on the separation of the functional bars (*d*). The distance that maximizes this reflection is around *d* = 24 μm (the case shown in Figure 2). It is noteworthy that for this distance the center-to-center bar separation *d* + 2(*w*/2) = 36 μm is close to a quarter of the operation wavelength (*λ*/4), a result that will be justified later on by the corresponding theoretical analysis.

### 2.3 Circular polarization response

Based on the response of the metasurface to linearly polarized waves, the response to circularly polarized waves can be either deduced via a change of basis from the linear one, { $\hat{x},\hat{y}$ }, to the circular, { ${\hat{e}}_{+},{\hat{e}}_{-}$ }, where ${\hat{e}}_{\pm }=\left(\hat{x}\pm i\hat{y}\right)/\sqrt{2}$ , or directly calculated in the circular polarization (CP) basis.

**Figure 3:**
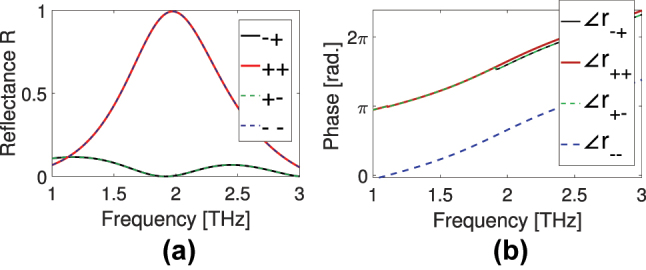
Numerically calculated reflection response of the metasurface to normally incident CP waves, (a) shows the reflectance (amplitudes-squared of reflection coefficients). Perfect co-polarized reflection is demonstrated in (a) (*R*_++_ in red, and *R*_−−_ in blue-dash) near 2 THz. *R*_−+_ is in black, *R*_+−_ is green dashed. (b) Shows the phases of the reflection coefficients.

Calculating the scattering response of our metasurface for right-handed circularly polarized (RCP/+) and left-handed circularly polarized (LCP/−) incident waves, we obtain what is shown in Figure 3 (note that the RCP here is considered proportional to ${\hat{e}}_{+}$ $\left({\hat{e}}_{-}\right)$ for wave-vector parallel (antiparallel) to the positive *z* direction (i.e. to $\hat{z}$ ); it is always denoted though by the subscript +). As can be seen in Figure 3(a), unlike what is observed in common chiral or non-chiral metasurfaces, where the largest (or the only) reflected CP component is the cross-polarized one, here perfect co-polarized reflections *R*_++_ (red) and *R*_−−_ (blue dashed) are achieved near 2 THz, while *R*_−+_ (black) and *R*_+−_ (green dashed) are suppressed (note again *R* = |*r*|^2^). This indicates the potential of our structure to act as helicity preserving mirror, even without the presence of a back-reflector (a useful capability of metasurfaces [44], [45]), i.e. leaving the rest of the spectrum available for, e.g., different functionalities. Perfect co-polarized reflections have the same magnitude squared for both RCP and LCP, but there is a phase difference of *π* between them as shown in Figure 3(b).

This helicity preserving reflection response of our structure can be exploited, among others, for the achievement of helicity-preserving cavities, valuable in molecular chirality sensing applications and enantiomer identification [29], [30]. The merit of our structure in molecular chirality sensing applications can be revealed also by the high values of the optical chirality obtained in the vicinity of the top/first bars, as shown in the Supplementary Information.

### 2.4 A physical interpretation of the two-bar system response

Considering the two horizontal metallic bars of our structure as two perpendicular abstract resonant electric dipoles (functioning as simple harmonic oscillators (uncoupled; owing to the structure symmetry) of resonance frequency *ω*_0_ and damping factor *γ*) separated by a vertical distance *d* and excited by a linearly polarized wave of the form **E** = **E**_**0**_ *e* ^*i*(*kz*−*ωt*)^ (*k* is the free-space wave number), we can achieve a simple physical picture of the structure response and understand its reflection properties. For simplicity we consider here the *z*-origin of the coordinate system at the first/top dipole position, while the second dipole is located at *z* = *d*. Moreover, we consider an auxiliary coordinate system { ${\hat{e}}_{1},{\hat{e}}_{2},\hat{z}$ }, with ${\hat{e}}_{1}$ $\left({\hat{e}}_{2}\right)$ unit-vectors along the direction of the first (second) dipole (for generality we consider the pair rotated by an angle *ϕ* in respect to *x* axis; see Figure 1(b); in the case of Figures 2 and 3, *ϕ* = *π*/4).

Using the simple oscillator model we can obtain the electric dipole moments of the two-bars (see Supplementary Information) as

(1) $$p_{1}\left(\omega \right)=\alpha \left(\omega \right)\left\langle {\hat{e}}_{1}|E\right\rangle {\hat{e}}_{1},$$

(2) $$p_{2}\left(\omega \right)=\alpha \left(\omega \right)\left\langle {\hat{e}}_{2}|Ee^{\mathrm{ikd}}\right\rangle {\hat{e}}_{2},$$

where **E** is the incident field at *z* = 0, ${\hat{e}}_{1}=\cos\left(\phi \right)\hat{x}+\sin\left(\phi \right)\hat{y}$ , ${\hat{e}}_{2}=-\sin\left(\phi \right)\hat{x}+\cos\left(\phi \right)\hat{y}$ and $\alpha \left(\omega \right)=\alpha_{0}\omega_{0}^{2}/\left(\omega_{0}^{2}-\omega^{2}-i\omega \gamma \right)$ is the electric polarizability of the abstract dipoles.

Considering the above dipole pair as the building block of an infinite (in *x*−*y* plane) uniform electric current sheet (i.e. our meta-surface), we can connect the dipole moments to the sheet electric current density, **j** = −*iω* **p**/*A*_*uc*_); then the total scattered/reflected field for normally incident waves can be written (at *z* = 0) as [53], [54]

(3) $$E_{r}=\frac{i\omega Z_{0}}{2A_{uc}}\left(p_{1}+p_{2}e^{-ik\left(-d\right)}\right),$$

where *Z*_0_ is the free space wave impedance and *A*_*uc*_ the unit cell area. If we consider the incident electric field polarized along *x*-direction, i.e. $E=E_{0}\hat{x}$ , by substituting Eqs. (1) and (2) into (3) and after some straightforward algebra we can find

(4) $$\begin{array}{ll} E_{r} & =\frac{i\omega Z_{0}}{2A_{uc}}\alpha \left(\omega \right)E_{0}\left[\left(\cos^{2}\phi +\sin^{2}\phi e^{2ikd}\right)\hat{x}\right.\\ & \;\left.+\cos\phi \sin\phi \left(1-e^{2ikd}\right)\hat{y}\right]. \end{array}$$

For *ϕ* = *π*/4, as in our case, Eq. (4) takes the form

(5) $$E_{r}=\frac{i\omega Z_{0}}{4A_{uc}}\alpha \left(\omega \right)E_{0}\left[\left(1+e^{2\mathrm{ikd}}\right)\hat{x}+\left(1-e^{2ikd}\right)\hat{y}\right].$$

Equation (5) shows that for *e* ^2*ikd*^ = −1, i.e. *d* = *λ*/4 (*λ* = 2*π*/*k*), the phase difference of the scattered waves by the two dipoles is such that the co-polarized reflection practically vanishes, while the cross-polarized reflection term gets its maximum value. This justifies the observed response in Figure 2, where the optimum distance for maximum cross-polarized reflection was found numerically to be close to *λ*/4.

To evaluate the scattered field for circular polarization one can use Eq. (3), writing it in the circular polarization basis, $\left\{{\hat{e}}_{+},{\hat{e}}_{-}\right\}$ , employing the formulas connecting the unit vectors ${\hat{e}}_{1},{\hat{e}}_{2}$ with the ${\hat{e}}_{+},{\hat{e}}_{-}$ (see Supplementary Information). Considering an incident RCP wave of the form $E=E_{0}{\hat{e}}_{+}$ , one can obtain for the reflected field (see Supplementary Information)

(6) $$E_{r}=\frac{i\omega Z_{0}}{4A_{uc}}\alpha \left(\omega \right)E_{0}\left[\left(1+e^{2\mathrm{ikd}}\right){\hat{e}}_{+}-e^{2i\phi }\left(1-e^{\mathrm{2ikd}}\right){\hat{e}}_{-}\right].$$

For *e* ^2*ikd*^ = −1, i.e. *d* = *λ*/4, as in our case, the ${\hat{e}}_{+}$ component of the reflected field, corresponding now to LCP wave (since the propagation is reversed), vanishes, indicating a pure RCP reflected wave:

(7) $$E_{r}=\frac{i\omega Z_{0}}{2A_{uc}}\alpha \left(\omega \right)E_{0}e^{2i\phi }{\hat{e}}_{-}.$$

We see in Eq. (7) that this helicity preserving component of the reflected wave acquires a geometric phase 2*ϕ* (Pancharatnam–Berry phase). This indicates the capacity of our structure to create phase gradient metasurfaces for the reflected CP waves, a capacity which is further discussed and demonstrated in Section 4.

### 2.5 Treatment of the system as two cascaded layers

The two-bar structure can be also treated as two separated layers of metasurfaces and analyzed using the transfer matrix method [47], [55], allowing for an accurate analytical description. Here we analyze its response to linearly polarized waves. We model the first bar as a single dipole oriented with *ϕ* = *π*/4, following the convention in Section 2.4. Here the reflection and transmission matrices (**R**_1_ and **T**_1_, respectively) can be written as **R**_1_ = *iωζ*_0_ *α*(*ω*)**J**/2, **T**_1_ = **I** + **R**_1_, where *ζ*_0_ = *Z*_0_/2*A*_*uc*_, **J** is 2-by-2 with all the elements equal to 1, and **I** is the identity matrix. The parameters entering in the Lorenz formula of *α*(*ω*) can be estimated by fitting the theoretical transmission or reflection to corresponding numerical results in terms of the magnitude and bandwidth. By inserting *ϕ* = 3*π*/4, we also obtain the reflection matrix **R**_2_ for the second bar which is orthogonal to the first one. The total reflection from the two-bar structure can be approximated as

(8) $$R_{\text{total}}\approx R_{1}+T_{1}T_{\text{delay}}R_{2}T_{\text{delay}}T_{1},$$

where **T**_delay_ is a diagonal matrix with diagonal elements *e* ^*ikd*^. At the resonant frequency *α*(*ω*_0_) takes the simple form of *α*_0_ *ω*_0_/(−*iγ*), and we arrive at the same condition of effective distance between the two dipoles *d* = *λ*/4 as in Section 2.4.

The presence of a substrate leads to a red shift of the dipole resonance, especially to the bar in contact with it, as will be discussed in Section 3. This effect, as well as the increased losses, can be taken into account by assigning a different resonant frequency $\omega_{0}^{\prime }$ and by adjusting *α*(*ω*). Further relevant discussion is in the Supplementary Information; results are also shown in Section 3.

## 3 Fabrication and experimental characterization

**Figure 4:**
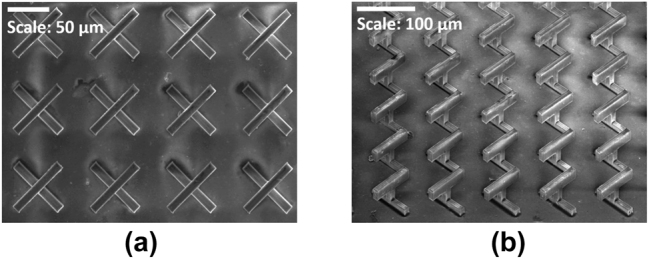
SEM images of the fabricated metallized metasurface structures with soda-lime-glass substrate, both in (a) top view, with a 50 μm scale bar; and (b) perspective view, with a corresponding 100 μm scale bar.

To fabricate the metasurface structure shown in Figure 1, we utilized direct laser writing (DLW) via multiphoton polymerization (MPP) [56], [57], [58], [59], followed by a selective post-metallization process known as silver electroless plating (SEP) [60]. The proposed metasurface was fabricated on both soda–lime–glass and silicon substrate, covering a total area of 3.4 × 3.4 mm^2^ (approximately 1,100 unit cells) in both cases. Details of the fabrication process and metallization are provided in the Supplementary Information and in [52]. Scanning electron microscope (SEM) images of the fabricated, metalized structures, showing both top and 45° tilted views, are presented in Figure 4. The measured geometric parameters of the metallic structures are as follows:

**Table:** 

Substrate	*P* [μm]	*l* [μm]	*w* [μm]	*d* [μm]
Glass	96.2	79.6	11.1	23.8
Silicon	97.1	80.2	11.2	24.4

Note though that the above parameters show some variations along the metasurface.

To characterize experimentally our structures, we employed THz time domain spectroscopy, sending a linearly *x*-polarized incident wave and measuring the co-polarized and cross-polarized reflection components under normal incidence. Details of the measurements are provided also in the Supplementary Information.

**Figure 5:**
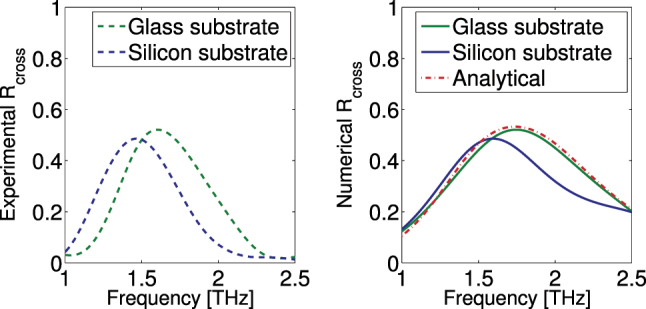
A comparison between normalized experimental (left, dashed), numerical (right, solid), and analytical (right, dash-dotted) results of the cross-polarized reflectance for LP incident waves, for structures fabricated on the glass substrate (green) and on the silicon substrate (blue). The analytical prediction (red) takes into account the losses and the red shift using the method in Section 2.5.

The measured cross-polarized reflection response of the fabricated structures is shown in Figure 5 along with corresponding numerical data. We have to note here that there are certain drawbacks in the fabrication process which do not allow an easy quantitative comparison between theory and experiment: Considerable losses are imported from the post-metallization process since SEP is achieved through a chemical procedure that covers the surface of the processed material with a thick (∼150 nm) layer of silver nanoparticles, enough to bypass the skin depth of silver in the studied frequencies, but of much lower conductivity than that of bulk silver. Measurements of the conductivity have given values as low as 10^4^ − 10^5^ S/m which seriously degrade the structure performance. Second, the bottom bar of the structure is attached onto the substrate (to achieve high stability and avoid deformations during SEP), and thus, the metamaterial is not homogeneously covered with silver nanoparticles. Third, there are slight variations in the dimensions of the top metallic bar compared to the bottom one, and there are also height (*d*) variations along the structure, which all affect negatively the reflection response, making also difficult an accurate normalization of the results. That’s why the raw experimental spectra from the metamaterial structure were divided by the corresponding experimental spectra of the incoming THz field.

Despite the above drawbacks, we observe a very good qualitative agreement between theory and experiment. A resonance in cross-polarized reflection, *R*_cross_, is observed also in the measurements, which is close to 1.6 THz for the structure made on the glass substrate and 1.5 THz for the silicon substrate.

Figure 5 also demonstrates the impact of the substrate on the structure reflection properties, observed in both simulations and experiment. The presence of the substrate leads to a red-shift of the cross-polarized reflection peak, which is stronger when the substrate permittivity is higher. Note that in the simulations we consider the relative permittivity of the substrate as *ɛ*_*r*_ = 6.45 + 1.66*i* for the soda–lime–glass [61] and *ɛ*_*r*_ = 11.683 + 0.04*i* for the silicon. The substrate thickness is taken as 1 μm to avoid the presence of Fabry–Perot resonances (which are eliminated in the THz-TDS procedure), while to account for the thicker substrate used in the experiment, the imaginary part of the substrate permittivity is increased in our simulations compared to the values of [61]; the conductivity of the metalized bars is taken as *σ* = 5.8 × 10^4^ S/m.

The substrate impact observed in Figure 5 is expected, given that the substrate red-shifts the electric dipole response of the bottom metallic bars (the ones touching the substrate; note that the resonance of a plasmonic particle red-shifts if the particle is placed in a dielectric environment, compared, e.g., to air).

## 4 Applications

**Figure 6:**
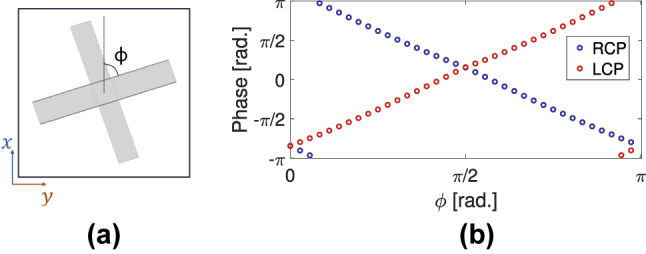
Phase response of the metasurfaces with the rotated unit cell. The schematic in (a) shows the two-bar structure rotated by an angle *ϕ*. (b) Shows the phase of the helicity-preserving reflection, numerically evaluated at 2 THz, over the rotation angle *ϕ*, for LCP (red) and RCP (blue) waves.

As shown in Section 2.4, the perfectly reflected helicity-preserving (co-polarized) component of our metasurface acquires a geometric (Pancharatnam–Berry) phase of 2*ϕ* if the meta-atom is rotated clock-wise by *ϕ* (Figure 6(a)). The sign of the geometric phase depends on the helicity of the incident CP waves. The phase of the co-polarized CP reflection, numerically evaluated at 2 THz, as we vary the rotation angle *ϕ*, is shown in Figure 6(b). The LCP case (red) and the RCP case (blue) exhibit opposite phase gradients as expected, with the slopes of the phase-change with *ϕ* being close to ±2. The reflection phase is the same for both helicities when *ϕ* = 0 or *π*/2. These two values of *ϕ* correspond to the scenarios when the two bars are aligned with the *x* and *y* axes, respectively. Meanwhile, although not shown here, the reflectance at 2 THz remains high as the unit cell rotates.

The phase response of Figure 6(b) allows the use of the two-bar meta-atom for the construction of beam steering and beam focusing metasurface structures, through design of proper supercells, as shown in the next subsections.

### 4.1 Beam steering

**Figure 7:**
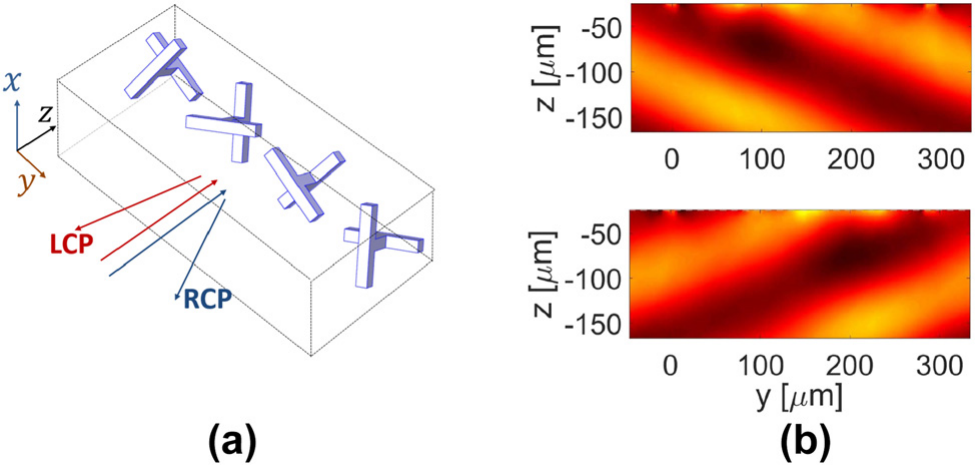
Numerical results of the reflected fields from the phase gradient structure. (a) Schematic of one supercell of four unit cells, illustrating that normally incident RCP (blue) or LCP (red) waves are reflected to opposite sides of the incidence normal, while preserving the helicity. Numerically calculated reflected electric field (*x* component) distribution for the structure of (a) are shown in (b) for LCP (upper panel) and RCP (lower panel) cases.

To achieve beam steering functionalities, a discretized linear phase gradient covering a range of 2*π* is required to couple the incident CP waves to higher-order diffraction modes, where the steering angle *θ*_*r*_ corresponding to the first reflected Floquet mode follows the generalized Snell’s law [1],

(9) $$\sin\theta_{r}-\sin\theta_{i}=\frac{\lambda }{2\pi n_{i}}\frac{d\varphi }{dx}$$

with *θ*_*i*_ = 0 for normal incidence, *n*_*i*_ = 1 as we consider free space, and d*φ*/d*x* = 2*π*/*a*_super_ is the required linear phase gradient (note that according to Eq. (7) *φ* = 2*ϕ*). *a*_super_ is the size of the supercell and we show the numerical results for a phase gradient metasurface with a supercell of four unit cells *a*_super_ = 4*a*. Therefore, a periodic spatial variation of the rotation angle *ϕ*(*x*) = *πx*/*a*_super_ is needed. We select the rotation angle of each unit cell based on the results shown in Figure 6(b) as: *ϕ*_1,2,3,4_ = *π*/12, *π*/3, 3*π*/5, 0.89*π*. Figure 7(a) is a schematic of the phase gradient structure, also illustrating that reflected helicity-preserving CP waves are expected to be steered to opposite sides of the incidence-normal for RCP and LCP waves, since the phase gradients are of opposite signs for the two cases as shown in Figure 6(b). The corresponding numerical results for both cases are shown in Figure 7(b) and (c). The reflected *E*_*x*_ field (with the field corresponding to the incident waves subtracted) is shown, whereas the *E*_*y*_ distribution shows the same pattern but with a shift of around ±*π*/2 (the sign depends on the helicity) in the phase. The center of the metasurface is positioned at *z* = 0 (on *y*-axis in the plots).

### 4.2 Focusing

Here we aim to achieve focusing at 2 THz along the *y* direction, for normally incident LCP waves. The required phase profile across the *y*-axis in this case can be expressed as $\varphi \left(y\right)=2\pi /\lambda \left(F-\sqrt{y^{2}+F^{2}}\right)$ [38], where we choose focal length *F* = 600 μm. To achieve a discretized form of the above phase profile we employed 19 unit cells along the horizontal (*y*) direction, and the structure is considered uniform in the vertical (*x*) direction. Open boundary is set in the *y* direction and periodic boundary in the *x* direction in the numerical model. Incident waves propagate along the positive *z*-axis.

**Figure 8:**
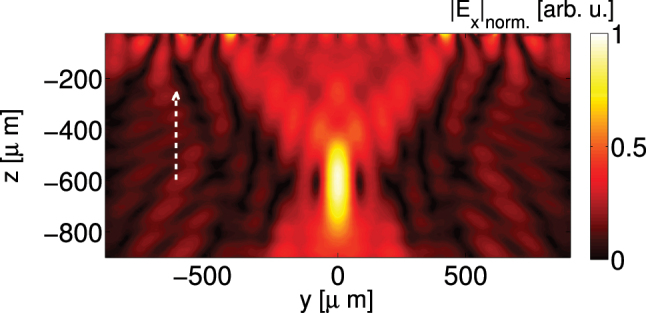
Simulated normalized reflected field distribution in the *y*–*z* plane showing a focal spot near *y* = 0, *z* = 600 μm; the structure is placed at *z* = 0. The field is normalized to it maximum value.

The simulated normalized reflected *E*_*x*_-field distribution at 2 THz in the *y*–*z* plane is shown in Figure 8. Normal incidence is shown by the white dashed arrow, and the field distribution is normalized to its maximum magnitude. As can be seen, the incident waves are focused with an approximate focal length of 600 μm.

## 5 Dielectric two-bar: scaling to the optical frequency range

Given that our metasurface response is mainly dictated by the structure geometry and that in the optical part of the spectrum displacement currents can undertake the role of conduction currents, it is interesting to examine if a fully dielectric nanoscale variant of our structure can bring its interesting and useful THz response to the optical region.

**Figure 9:**
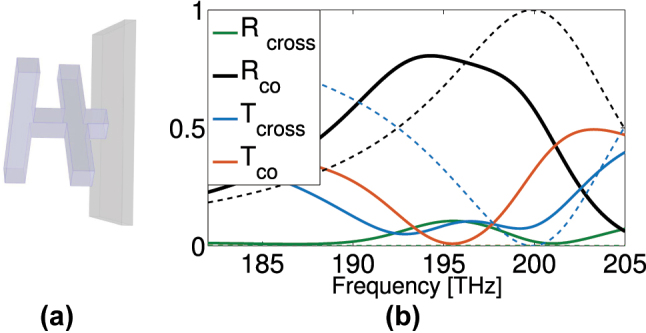
Dielectric two-bar unit cell. (a) Is the schematic showing a two-bar structure with a short connection to a thin substrate. (b) Is the reflectance and transmittance over frequency for both the optimized ideal unit cell (dashed) and the unit cell shown in (a) (solid).

For that we investigate the response of a dielectric two-bar metasurface made of silicon (of permittivity *ɛ*_*r*_ = 11.7) targeting operation at 200 THz. Figure 9 summarizes the achievable transmission and reflection response for a substrate-free structure (dashed lines) and for a structure with a dielectric substrate as shown in Figure 9(a) (solid lines), for CP incident waves. The parameters of the unit cell, following the notations in Figure 1, are *w*, *l*, *d* = 150, 1,100, 240 nm, and unit cell size *a* = 1,392 nm. The substrate is considered lossless with *ɛ*_*r*_ = 2.25 and thickness 80 nm. The substrate is semi-detached from the bars by the inclusion of a ‘leg’ section.

As can be seen in Figure 9(b), perfect reflection of the helicity preserving (co-polarized) CP wave component can be achieved also in the dielectric structure, at 200 THz, for the optimized, substrate-free structure. The inclusion of a thin substrate shifts the operating frequency to around 195 THz, while degrading the high-reflection performance (to a larger extent than in the THz metallic structure), in favor of transmittance and cross-polarized reflectance.

The dielectric two-bar structure shown in Figure 9 can be manufactured [62], [63] either in the form shown in Figure 9(a), employing direct laser writing, or by common lithographic techniques eliminating the vertical bar/leg and using a dielectric spacing layer to adjust the bars distance to the optimum values.

## 6 Conclusions

We have investigated a metasurface consisting of simple two-bar unit cells, and demonstrated its promising response in THz polarization-related applications: The metasurface can function as perfect cross-polarized reflector for linear wave polarization and as perfect helicity preserving mirror (valuable in chirality sensing applications) for circular polarization, without the use of any back reflector. This response was demonstrated numerically and analyzed through a simple oscillator model, while it was validated also by corresponding experimental results. The experimental structure has been fabricated using direct laser writing, followed by silver metallization via electroless plating, and characterized by THz-TDS.

The Pancharatnam–Berry phase induced by the rotation of our metasurface-unit-cell has been also explored, and we have numerically demonstrated its potential in beam steering and focusing applications. Lastly, we demonstrated scaling to the optical frequency range by a nanoscale all-dielectric analog of our structure. Numerical results have shown that the same helicity-preserving reflection behavior can be achieved by the all-dielectric metasurface.

## Supplementary Material

Supplementary Material Details
