# Supplementary material for: 3D-printed mirror-less helicity preserving metasurface “mirror” for THz applications
Source: Nanophotonics. 2025 Jun 9;14(23):4029–38. doi: 10.1515/nanoph-2024-0711 (PMC12617713; doi:10.1515/nanoph-2024-0711)
Supplement: Supplementary file 1 — Supplementary Material Details [file j_nanoph-2024-0711_suppl_001.pdf]

3D-printed mirror-less helicity preserving metasurface "mirror" for THz applications

J. Yan, I. Katsantonis, S. Papamakarios, P. Konstantakis, M. Loulakis, Th. Koschny, M. Farsari, S. Tzortzakis, M. Kafesaki

May 6, 2025

1 Theoretical and numerical analysis

1.1 The double oscillator model

In this section we analyze the most fundamental metasurface, with unit-cell made of a pair of perpendicular resonant metallic bars (at a distance d), as shown in Fig. 1. Considering these bars as two perpendicular harmonic oscillators which form the building block of an infinite (in x-y plane) electric current sheet), we derive the reflection coefficients for both linearly and circularly polarized waves. This analysis aims to provide a tool for the understanding of the underlying physics of our structure, for the identification of its capabilities, and for its optimization.

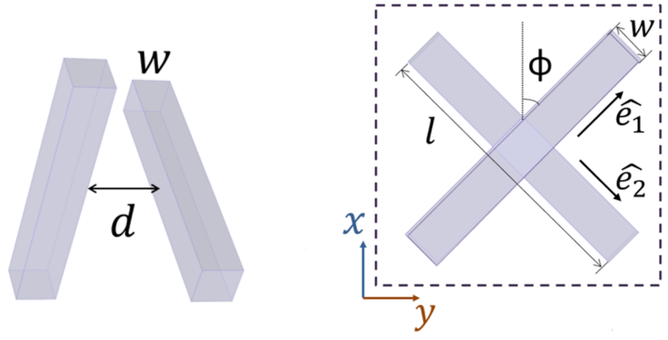

Figure 1: (a) Schematic of unit cell of our metasurface.

As shown in Figure 1, the top metal bar, considered at $z = 0$, is rotated by an angle ϕ relative to the x-axis. Considering an auxiliary coordinate system aligned with the two bars $(\hat{e}_1, \hat{e}_2, z)$, as discussed in the main text, and an applied electric field of the form $\mathbf{E}e^{i(kz-\omega t)}$, each bar can be described as a simple harmonic oscillator of charge $Q = -Ne$, resonance frequency ω_0 and damping factor γ. The charges under the impact of the applied field are displaced according to

$$\begin{aligned} \ddot{\mathbf{r}}_1 + \gamma\dot{\mathbf{r}}_1 + \omega_0^2\mathbf{r}_1 &= -(e/m)\langle\hat{e}_1|\mathbf{E}\rangle e^{-i\omega t}\hat{e}_1 \\ \ddot{\mathbf{r}}_2 + \gamma\dot{\mathbf{r}}_2 + \omega_0^2\mathbf{r}_2 &= -(e/m)\langle\hat{e}_2|\mathbf{E}\rangle e^{i(kd-\omega t)}\hat{e}_2 \end{aligned} \tag{1}$$

where $\mathbf{E}$ is the amplitude of the applied field. (Note that in the case of perpendicular "dipoles" and normal incidence the local field can be very well approximated by the external field, and there is no coupling between the dipoles.) Considering the time dependence of the solutions of Eqs. (1) to be of the form $e^{-i\omega t}$, and the dipole moments of the two bars given as $\mathbf{p}_i = -Ne\mathbf{r}_i \quad i = 1, 2$, the equations for the dipole moments (omitting the time dependence $e^{-i\omega t}$) take the form

$$\mathbf{p}_1(\omega) = \alpha(\omega)\langle\hat{e}_1|\mathbf{E}\rangle\hat{e}_1 \tag{2}$$

$$\mathbf{p}_2(\omega) = \alpha(\omega)\langle\hat{e}_2|\mathbf{E}e^{ikd}\rangle\hat{e}_2 \tag{3}$$

where $\hat{e}_1 = \cos(\phi)\hat{x} + \sin(\phi)\hat{y}$, $\hat{e}_2 = -\sin(\phi)\hat{x} + \cos(\phi)\hat{y}$, and $\alpha(\omega) = \alpha_0\omega_0^2/(\omega_0^2 - \omega^2 - i\omega\gamma)$ is the electric polarizability, with $\alpha_0 = Ne^2/m\omega_0^2$.

Considering the above dipole pair as the building block of an infinite (in $x - y$ plane) uniform electric current sheet, as discussed in the main text, we obtain for the total scattered/reflected field for normally incident waves (at $z = 0$) [RAT14, AAT16]

$$\mathbf{E}_r = \frac{i\omega Z_0}{2A_{uc}}(\mathbf{p}_1 + \mathbf{p}_2 e^{-ik(-d)}), \tag{4}$$

where Z_0 is the free space wave impedance and A_{uc} the unit cell area.

If we consider an incident electric field polarized along x-direction, i.e. $\mathbf{E} = E_0\hat{x}$, by substituting Eqs. (2) and (3) into Eq. (4) we can find

$$\mathbf{E}_r = \frac{i\omega Z_0}{2A_{uc}}\alpha(\omega)E_0[(\cos^2\phi + \sin^2\phi e^{2ikd})\hat{x} + \\ + \cos\phi\sin\phi(1 - e^{2ikd})\hat{y}] \tag{5}$$

If we consider the incident electric field to be polarized along y-direction, i.e. $\mathbf{E} = E_0\hat{y}$, we obtain

$$\mathbf{E}_r = \frac{i\omega Z_0}{2A_{uc}}\alpha(\omega)E_0[(\sin\phi\cos\phi(1 - e^{2ikd}))\hat{x} + \\ +(\sin^2\phi + \cos^2\phi e^{2ikd})\hat{y}] \tag{6}$$

Eqs. (5) and (6) give for the reflection matrix of the system

$$\begin{pmatrix} r_{xx} & r_{xy} \\ r_{yx} & r_{yy} \end{pmatrix} = i\omega\alpha(\omega)\zeta_0 \begin{pmatrix} \cos^2\phi + \sin^2\phi e^{2ikd} & \sin\phi\cos\phi(1 - e^{2ikd}) \\ \sin\phi\cos\phi(1 - e^{2ikd}) & \sin^2\phi + \cos^2\phi e^{2ikd} \end{pmatrix} \tag{7}$$

where in the reflection coefficients the first subscript denotes the reflected wave polarization and the second the incident polarization, and $\zeta_0 = Z_0/2A_{uc}$. We observe that independently of the angle ϕ $r_{xy} = r_{yx}$

For $2kd = \pi$ the above matrix is simplified as

$$\begin{pmatrix} r_{xx} & r_{xy} \\ r_{yx} & r_{yy} \end{pmatrix} = i\omega\alpha(\omega)\zeta_0 \begin{pmatrix} \cos(2\phi) & \sin(2\phi) \\ \sin(2\phi) & -\cos(2\phi) \end{pmatrix} \tag{8}$$

For $\phi = \pi/4$, as in our case, the diagonal elements of the reflection matrix vanish, with the off-diagonal getting their maximum value.

Here, we are also interested for circularly polarized waves; thus we calculate the circular waves reflection amplitudes, r_{++}, r_{--}, r_{+-} and r_{-+}, where again the first subscript indicates the reflected field polarization and the second subscript the incident field polarization, with the + indicating RCP waves and the - LCP waves. The reflection matrix for CP waves can be obtained by transforming the LP waves reflection matrix using the well known formula,

$$\begin{pmatrix} r_{++} & r_{-+} \\ r_{+-} & r_{--} \end{pmatrix} = \frac{1}{2}\begin{pmatrix} (r_{xx} - r_{yy}) + i(r_{xy} + r_{yx}) & (r_{xx} + r_{yy}) - i(r_{xy} - r_{yx}) \\ (r_{xx} + r_{yy}) + i(r_{xy} - r_{yx}) & (r_{xx} - r_{yy}) - i(r_{xy} + r_{yx}) \end{pmatrix}. \tag{9}$$

Substituting in the above formula the matrix of Eq. (8), we obtain

$$\begin{pmatrix} r_{++} & r_{-+} \\ r_{+-} & r_{--} \end{pmatrix} = i\omega\alpha(\omega)\zeta_0 \begin{pmatrix} e^{2i\phi} & 0 \\ 0 & e^{-2i\phi} \end{pmatrix}. \tag{10}$$

The two-dipole model allows us to qualitatively understand the electromagnetic response of the two bar metasurface as a function of the angle (ϕ) and distance (d) between the bars.

1.2 The transfer matrix based model

The transfer and reflection matrices of a single-layer metasurface, as discussed in Section 2.5 of the paper, can be expressed either based on the dipole model or based on simulated results of a single

layer. Modeling the meta-atom as a resonant dipole rotated by an angle ϕ relative to the x-axis, the reflection matrix takes a simplified form of Eq.(6)

$$\mathbf{R}_i = i\omega\alpha(\omega)\zeta_0 \begin{pmatrix} \cos^2(\phi) & \cos(\phi)\sin(\phi) \\ \cos(\phi)\sin(\phi) & \sin^2(\phi) \end{pmatrix} \tag{11}$$

where $i = 1$ corresponds to the first/top bar/layer and has $\phi = \pi/4$, and $i = 2$ corresponds to the second bar/layer and has $\phi = 3\pi/4$. In order to take into account the losses and the effect of the substrate as in the experimental analysis, we adjust the values of α_0 ($\alpha(\omega)$ is proportional to α_0 as discussed in the previous section) and the resonant frequencies of the bars. As explained previously, α_0 is proportional to N (representing the amount of charges in the dipole oscillator), and α_0 reduces as the losses increase (there is a reduction in the metal conductivity of the fabricated sample, and the substrate loss contribute to the total loss). In addition, the resonant frequency ω_0 of the bar connected to the substrate shifts to a lower value. We obtained the analytical result fitted to the experimental results in Figure 5 of the paper through adjusting the afore-mentioned parameters.

Apart from the method discussed in Section 2.5 of the manuscript which sums the reflections, a more systematic approach merely based on matrix multiplications can be adopted [RG17].

1.3 Optical chirality

An object that can not be superimposed to its mirror image is called chiral, with its two mirror-image-related forms known as enantiomers. Chiral media, like most of the natural biomolecules, absorb to a different degree left-handed circularly polarized (LCP) waves than right-handed circularly polarized waves (RCP). This absorption (A) difference, i.e. $A_{LCP} - A_{RCP}$, is known as circular dichroism (CD) and it is used to identify the enantiomeric form of a chiral object. Calculating the absorption of LCP and RCP waves by a thin chiral layer [KDS+22, TC10], one can find that CD $= 4c\mathrm{Im}(\kappa)(\omega/2c^2)\mathrm{Im}(\mathbf{E}\cdot\mathbf{H}^*) = 4c\mathrm{Im}(\kappa)C$, where κ is the chirality (Pasteur) parameter of the chiral medium, which is opposite for the two enantiomers, ω is the wave angular frequency and c the vacuum light velocity. The quantity

$$C = C(\mathbf{r}) = -\frac{\omega}{2c^2}\mathrm{Im}(\mathbf{E}(\mathbf{r})\cdot\mathbf{H}^*(\mathbf{r})). \tag{12}$$

is a function of the local electric and magnetic field (without the presence of the chiral sample) and is called optical chirality. Optical chirality quantifies the impact on the CD of the electromagnetic environment where the chiral medium is placed. Fields of optical chirality larger than that of circularly polarized waves of the same frequency are characterized as super-chiral fields.

Own to the very small values of κ in natural chiral media (of the order of $10^{-4} - 10^{-5}$), circular dichroism response in natural chiral media is very weak and it is difficult to be measured and used for quantification purposes. A way to increase this differential absorption (as well as differential emission and any other excitation process that involves absorption or emission) is to combine the chiral media with super-chiral near fields, e.g. to put chiral media in the vicinity of super-chiral (of high C) fields.

Equation (12), being the scalar product of electric field and of $\pi/2$ phase shifted magnetic field, suggests that maximization of C requires large electric and magnetic fields with a $\pi/2$ phase shift between them, conditions that can be achieved through interaction of a wave with properly designed structures.

Given the importance of molecular chirality sensing and of enantioselective processes, especially in biology and pharmacology, the attempts of generation of super-chiral fields has been an important topic in the photonics research, especially in nanophotonics. While enhancement of local optical chirality has been already demonstrated in several works involving nanoscale metasurfaces, it is still a challenge to achieve large average C over large areas (in most of the cases the local C values variate over the unit cell from positive to negative, giving moderate average values). Here we show that our simple design can provide not only a high enhancement of the optical chirality, but also large continuous areas where fields with strong optical chirality (of the same sign) are located and can be accessed by chiral bio-molecules.

In Fig. 2 we show the optical chirality evaluated on two planes close to the free-standing ideal two-bar metasurface, at 2 THz, plotted over one unit cell area. The results are normalized to the optical chirality of the incident circularly polarized wave. Closer to the metasurface, 6 μm from the top surface of the first bar (Fig. 2(a)), there is stronger enhancement near the center of the meta-atom.

Slightly farther apart, at 16 μm from the meta-atom (Fig. 2(b)), the normalized optical chirality is more homogeneous although with lower values (still higher than unity). It should be noted that for both cases, the sign of the optical chirality does not change across the unit-cell area.

Given its largely uniform optical chirality, one way to exploit our structure in chirality sensing applications is to place the chiral molecules at the plane of larger C and to perform the sensing in a photoluminescence experiment, monitoring enantioselective photoluminescence, an approach though not suitable for the THz region. The most promising approach though is to use our handedness-preserving mirror for formation of chiral cavities, by placing two such mirrors as to form a Fabry-Perot-type resonator (slightly detuned from the perfect reflection condition). Preliminary simulations in such type of resonator have shown a 10-fold C enhancement in a large part of the volume of the cavity. Detailed analysis and quantification of the sensing capabilities of our structures though is beyond the scope of the current work.

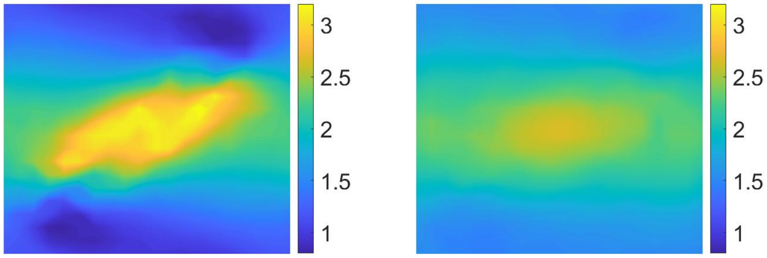

(a) (b)

Figure 2: Optical chirality evaluated at 6 μm (a) and 16 μm (b) from the top bar, normalized to that of the incident wave at 2 THz. The distribution is plotted over the surface area of one unit cell.

2 THz metasurface fabrication

For the fabrication of the two-bar resonator metasurface we use multi-photon lithography (MPL), which is a true 3D printing fabrication process relying on Direct Laser Writing (DLW). This layer-by-layer approach enables the process of photosensitive materials using non-linear optical effects, resulting in high aspect ratio structures using a mask-less procedure with noticeable repeatability, reaching to a resolution beyond the diffraction limit (below a few tens of nm).

The photoresist that was used for this specific application is a hybrid material combining 2-(dimethylamino)ethyl methacrylate (DMAEMA) for the organic part and zirconium n-propoxide (ZPO) for the inorganic part. ZPO is used for its polymeric properties and offers high mechanical stability, while adding DMAEMA in proportion of 30% to ZPO results in formation of metal-binding moieties on processed material, enabling electroless silver plating to get the final metal-covered structure. The photoresist contains also 4,4'-bis(diethylamino) benzophenone (Michler's ketone, Sigma-Aldrich) as photoinitiator, in ratio 1% w/w to the monomers. Development of the phororesist, as well as detailed steps for the post-process metallization can be found in the supplementary file in ref. [PTK+24, KMK+23]. Before the photoresist was processed, it was dropcasted on Si and thick glass substrates that were previously processed using a solution of methacryloxypropyltrimethoxysilane (MAPTMS) and dichloromethane (DCM) at 1:80 volume ratio in order to create a thin layer of bonding molecules between the substrate and polymerised photoresist, following the procedure described in ref.[LND+22].

In order to use multiphoton lithography as a fabrication tool, we used our in-home setup which is based on triggering multiphoton absorption with a source of a femtosecond laser beam, tightly focused through a high numerical aperture objective lens inside a very small (focal) volume of the photoresist. The laser source is a fiber femtosecond system operating at 780 nm, with pulse duration of 150 fs and repetition rate of 80 MHz (FemtoFiber ultra 780, Toptica Photonics AG). An acousto-optical modulator (AOM) (MTS40-A3-750.850, AA Opto Electronics) is used as a shutter and attenuator, while the printing process is controlled by a 2D galvo head (Hurryscan II 10, Scanlab) that consists

galvanometric mirrors that guide the beam in X and Y direction inside the photoresist. Then, the beam is focused through a plan-apochromatic objective lens with 20X magnification (ZEISS) and N.A. of 0.4, while the sample is placed on a XYZ axis system on the top of three linear translation stages (Physik Instrumente). The printing process is controlled using an advanced fabrication software (Arachne, Biomimetic), which enables the adjustment of fabrication power and speed, as well as translating the CAD design into a gcode directly, and fixing the tilt correction of the sample.

The fabrication starts from the bottom (attached onto the substrate) of the structure, moving layer by layer to the top of it with a slicing distance of 1 μm and 0.5 μm hatching distance. Creation of stable and repeatable structures was achieved by setting power at 80 mW (measured with a powermeter before the galvo head), and the mark speed of the galvanometric mirrors at 8 mm/s to minimize the fabrication time. Using stitching process we were able to fabricate a total surface of 3.4 mm X 3.4 mm (Fig. 3(a)), keeping the size of the unit cell constant (see Fig. 3(b)).

After the fabrication process, the sample was immersed in 4-methyl- 2-pentanone and subsequently rinsed in 2-propanol, in order to remove the excess unprocessed material. Then, a metallic coating using silver nanoparticles was selectively placed on the processed material, following the protocol described in Ref.[PTK+24]. SEM images of the silver nanoparticles formed on the surface of the polymer are depicted in Fig. 3(d)). This process allows to create metallo-dielectric structures, selectively metallized keeping the substrate intact from metallic elements (Fig. 3(c)).

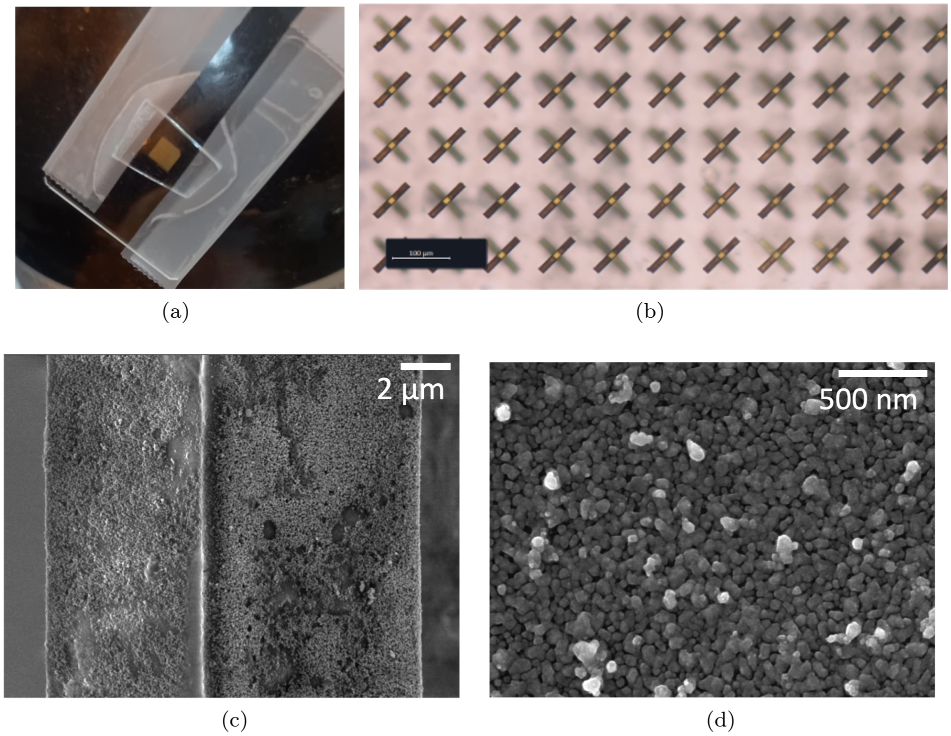

Figure 3: Experimental results of the fabricated sample: (a) image of the final sample, (b) optical microscope image of the metallized structure onto glass substrate, (c) SEM image of the silver coating formed on the surface of the structured polymer selectively (more specific, here we present the connection bar between bottom and top bar), and (d) high magnification SEM image showing the silver nanoparticles.

3 Experimental electromagnetic characterization

In order to characterize our metasurface, a terahertz time-domain spectroscopy (THz-TDS) system was employed, utilizing photoconductive antennas (TOPTICA TeraFlash pro). Our setup has been refined with in-house custom modifications, enabling us to acquire measurements in reflection configuration, under normal incidence. A graphical illustration of the experimental setup that we used is shown in Fig. 4, capable of generating a broadband THz pulse with a bandwidth greater than 5 THz. The THz emitter outputs a beam that is linearly polarized along the x-axis, and is collimated and focused from a pair of off-axis parabolic mirrors (PM1 and PM2). A THz wire-grid polarizer (POL1) is also inserted along the beam path, to ensure a well-defined polarization direction along the x-axis. Following the initial polarizer, a piece of high-resistivity silicon is placed, acting as a beam-splitter (BS) in the THz regime. The beam interacts with the metasurface sample, placed on an XY translation stage, under normal incidence. As a result, the reflected beam, "experiences" a rotation of its polarization axis, which is now parallel to the y-axis. Then, the beam is driven to a second wire-grid polarizer (POL2) whith axis parallel to the y axis, after being reflected from the BS. Finally, the beam is collected from a a pair of two off-axis parabolic mirrors (PM3 and PM4) and arrives at the THz receiver.

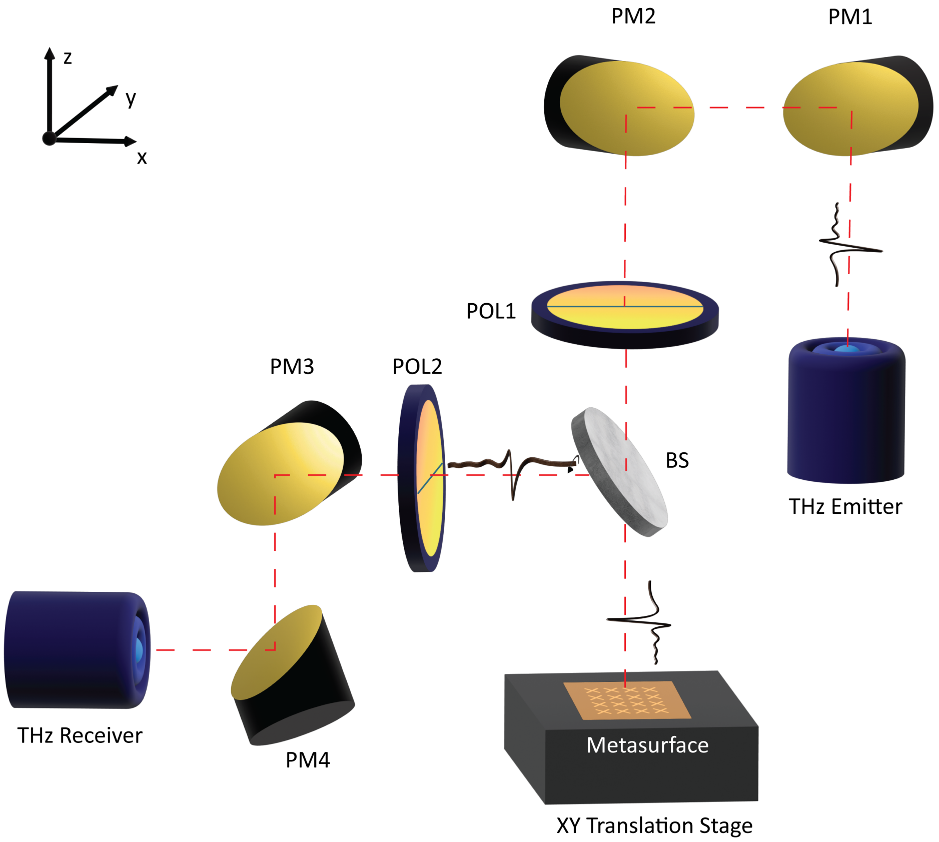

Figure 4: Schematic representation of the experimental electromagnetic characterization (THz-TDS) setup. PM1,2,3,4: Off-axis parabolic mirrors. POL1,2: Wire grid polarizers, BS: Beam-splitter.

References

[AAT16] Viktar Asadchy, Mohammad Albooyeh, and Sergei Tretyakov. Optical metamirror: all-dielectric frequency-selective mirror with fully controllable reflection phase. *JOURNAL OF THE OPTICAL SOCIETY OF AMERICA B-OPTICAL PHYSICS*, 33(2):A16–A20, FEB 1 2016.

[KDS+22] Ioannis Katsantonis, Sotiris Droulias, Costas M Soukoulis, Eleftherios N Economou, T Peter Rakitzis, and Maria Kafesaki. Chirality sensing employing parity-time-symmetric and other resonant gain-loss optical systems. *Physical Review B*, 105(17):174112, 2022.

[KMK+23] Ioannis Katsantonis, Maria Manousidaki, Anastasios D Koulouklidis, Christina Daskalaki, Ioannis Spanos, Constantinos Kerantzopoulos, Anna C Tasolamprou, Costas M Soukoulis, Eleftherios N Economou, Stelios Tzortzakis, et al. Strong and broadband pure optical activity in 3d printed thz chiral metamaterials. *Advanced Optical Materials*, 11(18):2300238, 2023.

[LND+22] D Ladika, G Noirbent, F Dumur, D Gigmes, A Mourka, GD Barmparis, M Farsari, and D Gray. Synthesis and application of triphenylamine-based aldehydes as photo-initiators for multi-photon lithography. *Applied Physics A*, 128(9):745, 2022.

[PTK+24] Savvas Papamakarios, Odysseas Tsilipakos, Ioannis Katsantonis, Anastasios D Koulouklidis, Maria Manousidaki, Gordon Zyla, Christina Daskalaki, Stelios Tzortzakis, Maria Kafesaki, and Maria Farsari. Cactus-like metamaterial structures for electromagnetically induced transparency at thz frequencies. *ACS Photonics*, 2024.

[RAT14] Younes Ra'di, Viktar S. Asadchy, and Sergei A. Tretyakov. Tailoring reflections from thin composite metamirrors. *IEEE TRANSACTIONS ON ANTENNAS AND PROPAGATION*, 62(7):3749–3760, JUL 2014.

[RG17] Amin Ranjbar and Anthony Grbic. Analysis and synthesis of cascaded metasurfaces using wave matrices. *Physical Review B*, 95(20):205114, 2017.

[TC10] Yiqiao Tang and Adam E Cohen. Optical chirality and its interaction with matter. *Physical review letters*, 104(16):163901, 2010.
